# Bacteriophages as Alternatives to Antibiotics in Clinical Care

**DOI:** 10.3390/antibiotics8030138

**Published:** 2019-09-04

**Authors:** Danitza Romero-Calle, Raquel Guimarães Benevides, Aristóteles Góes-Neto, Craig Billington

**Affiliations:** 1 Postgraduate Program in Biotechnology, State University of Feira de Santana (UEFS), Av. Transnordestina S/N, Feira de Santana-BA 44036-900, Brazil; 2 Health & Environment Group, Institute of Environmental Sciences and Research, PO Box 29-181, Christchurch 8540, New Zealand

**Keywords:** bacteriophages, clinical trials, antibiotic resistance, infectious disease, phage therapy

## Abstract

Antimicrobial resistance is increasing despite new treatments being employed. With a decrease in the discovery rate of novel antibiotics, this threatens to take humankind back to a “pre-antibiotic era” of clinical care. Bacteriophages (phages) are one of the most promising alternatives to antibiotics for clinical use. Although more than a century of mostly ad-hoc phage therapy has involved substantial clinical experimentation, a lack of both regulatory guidance standards and effective execution of clinical trials has meant that therapy for infectious bacterial diseases has yet to be widely adopted. However, several recent case studies and clinical trials show promise in addressing these concerns. With the antibiotic resistance crisis and urgent search for alternative clinical treatments for bacterial infections, phage therapy may soon fulfill its long-held promise. This review reports on the applications of phage therapy for various infectious diseases, phage pharmacology, immunological responses to phages, legal concerns, and the potential benefits and disadvantages of this novel treatment.

## 1. Introduction

There are approximately 10^30-31^ bacteriophages (phages) in the biosphere [1,2], which is estimated to be 10-fold higher than the total number of bacterial cells [3]. Phages are also an inherent part of the human microbiome, and so are usually well-tolerated when used in phage therapy [4,5,6]. Phages are one of the most promising alternatives to antibiotics, which can be used for medicine, agriculture, and related fields [7]. The evolution of multidrug-resistant and pan-drug-resistant bacteria poses a real threat to the control of infectious diseases globally, so it is urgent to have new therapeutic tools available. The United States National Institutes of Health have stated that phages are promising tools for combatting microbial resistance [8].

A post-antibiotic era in which minor injuries and common infections can kill because of the lack of drugs or their ineffectiveness is nowadays not an apocalyptic fantasy, but a real 21st-century threat. For example, ESKAPE organisms (*Enterococcus faecium*, *Staphylococcus aureus*, *Klebsiella pneumoniae*, *Acinetobacter baumannii*, *Pseudomonas aeruginosa,* and *Enterobacter* spp.) are extremely resistant to multiple antimicrobial agents [9] and are a serious challenge in medicine today. On the other hand, there historically has been no fit for purpose regulatory framework to deal with novel flexible and sustainable therapeutic approaches such as phages. For phages, this includes oversight of the setup and approval of adequate clinical trials, so as a result, there is no standard protocol for phage therapy.

In this review, we summarize the phage therapy clinical trials that have shown promising results in patients. We cover several diseases, immunological responses to phages, phage pharmacology, legal concerns about phage therapy, phage genetic modification, and a description of the advantages and disadvantages of phage therapy when compared to conventional treatments with antibiotics.

## 2. Phage Biology

Viruses that infect bacteria and *Achaea* are called phages, which have no machinery for generating energy and no ribosomes for making proteins. They are obligate bacterial parasites that carry all the genetic information required to undertake their reproduction in an appropriate host. The genome size of phages varies from a few thousand base pairs up to 498 kilobase pairs in phage G, which is the largest phage sequenced to date [10]. Most phages have a high level of host specificity (though some are broad in range), high durability in natural systems, and the inherent potential to reproduce rapidly in an appropriate host. They can be found associated with a great diversity of bacterial species in any natural ecosystem [11].

Phages can be characterized by their size and shape into three general groups: icosahedron, filamentous, and complex. Members of these groups may contain nucleic acid of various types including single-stranded DNA (ssDNA), double-stranded DNA (dsDNA), single-stranded RNA (ssRNA), or double-stranded RNA (dsRNA). Phages can be further classified with respect to their actions that follow infection of the bacterial cell. Virulent bacteriophages reproduce immediately and induce lysis of the cell to enable progeny release, whereas temperate phages insert their genetic material into the host genome or accessory elements, where they reproduce with the host until triggered to enter the lytic pathway as observed for virulent phages [12].

Virulent tailed phages of the Caudovirales order have been the best described for phage therapeutic applications. Within this group, the Myoviridae have a large capsid head and contractile tail, the Siphoviridae have a relatively small capsid and a long flexible non-contractile tail, and the Podoviridae have a small capsid head and short tail [13]. The virulent tailed phages follow a lytic cycle that begins with the specific attachment of phage anti-receptors to host cell surface receptor molecules. This interaction is often two-step, with an initial reversible phase and then irreversible phase. Once irreversibly bound, enzymes degrade the cell wall and the genetic material is ejected into the cell with (usually) the assistance of processive host enzymes. Once transcribed, the phage genome begins to redirect the host cell metabolism including DNA replication and protein biosynthesis to the reproduction of viral nucleic acid and proteins. Often, the host genome is degraded during this process. Once complete daughter viral particles are assembled, cell lysis is initiated to release the particles. Bacterial lysis is triggered by late encoded phage proteins including holins (to permeabilize the inner cell membrane) and endolysins (to degrade the peptidoglycan) with the loss of cell wall integrity causing lysis due to osmotic differential [14].

### Specificity

Host specificity (range) of phages is variable, with some phages infecting multiple species and others only growing on one known isolate. However, their specificity is much higher than that of antibiotics. The phage host cell surface receptors and antiviral defense mechanisms (genetic and physical) are the main properties that determine specificity. For some highly conserved species, a single phage can kill the majority of strains (e.g., phage P100 infects >90% *Listeria monocytogenes* isolates tested [15]). Phages that propagate on species with high clonal diversity (e.g., *Pseudomonas aeruginosa*) typically only kill a small cohort of strains [16].

Establishment of phage banks or training (in vitro evolution) of phages to become more active and to elicit less bacterial resistance against the infecting bacterial strain can be valid strategies to overcome limited host specificity for targeted phage therapy [16]. This strategy likely works best for chronic infections where the target bacterium is well characterized. In order to treat acute infections, phage cocktails including phages that together span the whole spectrum of potential strains are proposed. However, the research and resources needed for the production of suitable and stable multi-component cocktails are disadvantages of this approach. An alternative approach is to use phage lytic enzymes (endolysins), which show broader host specificity at the genus and species level. Endolysins have been the subject of a recent review by our group [17], so are not discussed further here.

Antibiotics typically kill a broad-spectrum of either Gram-positive and Gram-negative bacteria including benign flora, which is increasingly considered to be non-desirable due to their adverse effects on the whole microbiota and potential to spread antibiotic resistance [18,19]. Phage therapy meets these challenges by its superior specificity and ability to treat drug-resistant isolates.

## 3. Phage Pharmacology

The pharmacology of phages necessitates the study of interactions between phages and bacteria as well as interactions between phages and body tissues [14]. Successful and safe phage therapy involves the effective control of phage–host interactions involving two fundamental components: pharmacodynamics and pharmacokinetics [20].

### 3.1. Pharmacodynamics

Pharmacodynamics is the study of the interaction of drugs with their receptors, the transduction systems to which they are related, and the changes in cells, organs, and the whole organism. The drugs’ impact on the body can either be positive, thus maintaining or restoring health, or negative such as causing toxic side effects [20].

Phages can be applied via active or passive therapeutic strategies. In active treatment regimes, phages are introduced at low concentrations relative to the bacteria concentration and therapy relies on the production and release of progeny phages to infect all bacteria. Active treatments with phages are considered to have features of automated dosing and to mimic the bodies’ homeostatic mechanism better than standard pharmaceuticals through the targeted killing of bacteria and phage production at actual sites of infection rather than systemically [21]. In contrast, passive phage treatment relies on single, or multiple rounds of sufficient phage concentrations to infect all target bacteria.

Compared to antibiotics, only a single phage is required to kill a single bacterium and so fewer units are required per treatment. Phages also do not dissociate from bacterial targets once irreversibly adsorbed. However, multiple phages may adsorb to individual bacteria. For these reasons, it is important to understand the concepts of multiplicity of infection (MOI), which is the ratio of phage infections per bacteria, and MOI _input_, which is the number of phages that are administered per cell. The killing titer is another concept that can be used to guide phage therapy and is the number of effective bactericidal phage particles delivered (c.f. the number of plaque-based phage counts) [14,20,21,22,23]. Failure to recognize the special requirements of phage pharmacodynamics could result in compromises to phage therapy efficacy [20].

The degradation of phages by antibodies and other aspects of the immune system do not lead to the production and accumulation of toxic by-products. The low toxicity of phages is a consequence of their composition which, for tailed phages, is entirely protein and nucleic acid. As a result, phage therapy can be considered comparatively physiologically benign when compared to standard antibiotic therapies.

### 3.2. Pharmacokinetics

Pharmacokinetics describes the absorption, distribution, metabolism, and excretion of a drug. Absorption and distribution of the drug require its movement throughout the body, at first to the blood and then beyond the blood into specific tissues or compartments where the drug may accumulate at different densities [20]. Phage pharmacokinetics are also influenced by decay and proliferation as a result of the self-replication of bacteriophages.

The route of administration for phages will also affect in situ pharmacokinetics. In clinical cases, phages are frequently delivered by parenteral administration with oral dosing, topical application, and aerosolization also common. Data on the relative effectiveness of these approaches is largely drawn from animal studies. For instance, intramuscular, intraperitoneal, and subcutaneous injection of a phage cocktail were compared for efficacy in treating a *P. aeruginosa* in a murine burn model where intraperitoneal injection was found to be the most effective, most likely due to the delivery of higher numbers of phages more quickly and for a greater sustained period than other routes [24]. When using oral phage dosing in mice, the addition of 0.025% CaCO_3_ was found to effectively protect the phage from stomach acids and deliver the phage to the upper and lower gastrointestinal tract where they reduced numbers of the targeted *E. coli* O157:H7 [25]. When treating *Burkholderia* infections induced in mice, the aerosolization of phages was found to be superior to intraperitoneal injection [26]. Some advantages and disadvantages of the administration routes are shown in Table 1.

In vitro studies of phage pharmacokinetics using mathematical models do not necessarily reflect the in vivo phage kinetics observed. For instance, phage T4 was reported to not replicate in vitro at host concentrations below 10^4^ per mL, but evidence suggests that this is possible in murine models [27]. Phage feeding experiments in animals and humans frequently report irregular shedding and the passage of high percentages (up to 90% administered) of phages in feces [27]. The failure of many phage therapy experiments has been related to a poor understanding of phage pharmacokinetics, for instance, when dosing relies too much on the self-replicating nature of phages [20].

Phage lytic enzymes (endolysins) can also be used for therapy, but their kinetics are more similar to conventional treatments. For example, Jun et al. [28] determined that a *Staphylococcus aureus* specific endolysin had a half-life between 0.04 and 0.38 h after intravenous administration in healthy volunteers. The decay kinetics of this endolysin is likely explained by the presence of plasma proteases. Other endolysins have demonstrated a longer half-life such as 11.3 h for CF-301 and 5.2–5.6 h (for 30 and 60 mg/kg, respectively) for P128 [29,30].

Toxin (e.g., endotoxin) release due to significant bacterial cell lysis could potentially trigger septic shock during phage therapy. However, antibiotics like amikacin, cefoxitin, and imipenem have been shown to induce higher amounts of released endotoxin than coliphages [31]. The increase in endotoxin produced after 180 min incubation of *E. coli* LM33 was 3.8-fold with phage LM33_P1, 5.5-fold with amikacin, 8.7-fold with cefoxitin, and 30-fold with imipenem. With *E. coli* strain 536, there was a 19.8-fold increase in endotoxin with amikacin, 29.9-fold with phage 536_P1, 53.7-fold with imipenem, and 125.1-fold with ceftriaxone [31].

So, whilst less of an issue than for most conventional antibiotics, high fragmentation of the cell wall must be minimized with either phage or phage endolysin therapies to prevent an increase in pro-inflammatory cytokines [19,32]. To address this potential issue, several groups have proposed genetically engineering phages to prevent or reduce cell lysis, whilst still causing cell death by mechanisms such as degrading the host genome (see Section 7 and [33]).

## 4. Role of the Immune Response in Phage Therapy

Phages can potentially trigger innate and adaptive immune cells that may influence the success of phage therapy. Three major fields of phage-immune interaction can be discerned. First, involving immune recognition via pattern recognition receptor (PRR), which is a means for the recruitment of phagocytes to the infection site [34]. Phages can mediate the activation of innate immune cells when PRR recognizes phage-derived DNA and RNA. The extent of immune activation will differ depending on the phage type, the phage dose, and in vivo nucleic acid synthetic activity. 

Second, promoted phage-neutralizing antibodies can hamper therapeutic success and this effect can increase with repeated administration [35]. Antibody induction against phages is considered to be highly variable, thus immunogenicity should be considered during phage screening prior to phage therapy. There are several externally presenting proteins on phages such as Hoc, which can potentially induce such an immune response [36,37,38]. Strategies to avoid phage-induced neutralizing antibody formation include refining dose concentrations, the use of low-multi-dose regimes, or low-dose passive therapy approaches.

Third, the inhibitory effect of humoral (adaptive) immunity and anti-phage antibody production on phages in the mammalian system is broadly known. Effects seem dose-dependent, with only high doses for long periods inducing specific responses. For instance, Majewska [39] developed a long-term study of antibody induction (IgM, IgG, secretory IgA) in mice fed T4 phage orally at high doses (10^9^ PFU/mL drinking water). No effect was noted in the first two weeks, then in weeks 3–5, there was an increase in blood serum IgG. IgM did not increase until IgG began increasing, while IgA did not increase until days 63–79, but when it reached its maximum, no phage was found in the mouse feces. Increased IgA concentrations antagonized the gut transit of active phage and phage resistant hosts dominated the gut flora by day 92. However, IgA was rapidly cleared after phage withdrawal [39]. A similar study determined the immunological response of *Pseudomonas* phage F8 and T4 treatment in a murine systemic inflammatory response syndrome (SIR) model. The primary (IgM) and the secondary (IgG) responses inhibited the phages, and phage concentration in the spleen was significantly decreased [40].

Human trials in 26 patients with immunodeficiency diseases were undertaken to evaluate immunologic responses to phage ϕX174. An intravenous dose of 10^9^ PFU/kg body weight was given, and the phage titer measured in blood. No antibody response was detected in eight cases of infantile X-linked agammaglobulinemia with circulating phages present for up to 11 days. The other 18 patients produced antibodies and phages were cleared from circulation within four days. Ten of these patients showed the IgM antibody, and eight patients produced both IgM and IgG [41]. Other work using ϕX174 [42] has demonstrated that repeated (up to quaternary) dosing of phages does not lead to serious adverse reactions.

It is currently not well understood if anti-phage antibodies could prevent bacterial resistance development to phages and if the pre-existing immunity to natural phages could affect phage therapy. Furthermore, there is no clear information about the impact of phage-specific factors on phage clearance mechanisms. There are also gaps in our understanding of the clinical relevance of the phage immune interaction. Nevertheless, the immunogenicity of phages itself does not seem to represent a significant safety risk for patients. Reports about immune effects in clinical studies using virulent phages are limited. The introduction of validated in vitro and in vivo methods to determine the comparability of immune effects of different phages and phage combinations would be indispensable. This would allow for valid conclusions on the value of immune-based parameters for the selection of phages, identification of responsive patient populations, exchangeability of phages, and the importance of individualized phage cocktails [33]. The engineering of phages to make them less immunogenic is also an area of active research (see Section 7).

## 5. Resistance to Phages

An important consideration for phage therapy is the potential for bacterial resistance. Phage-resistant bacteria have been noted in up to 80% of studies targeting the intestines and 50% of studies using sepsis models, with phage-resistant variants also observed in human studies [43].

As with resistance to classical antibiotics, spontaneous resistance to phages may occur through a number of mechanisms. For example, the cell surface target receptor(s) may not be expressed or become mutated, thus causing a complete loss of adsorption or decreased adsorption. This is a limitation of both phage and conventional antibiotic therapy. For both approaches, knowing the receptor site(s), their stability, and conservation across strains will help with the mitigation of resistance. 

Acquired resistance is another area that requires investigation for both therapeutic approaches. Accessory genetic elements such as plasmids, temperate phages, and mobile genetic islands can carry genes coding for resistance to antibiotics. For phages, acquired resistance can encompass CRISPR-Cas systems [33], immunity proteins produced by temperate phages (though rare) and the acquisition of DNA restriction-modification systems.

A key advantage of phage therapy over conventional treatments for the avoidance of resistance development is the deployment of phage cocktails. The use of several phages, each targeting different receptors and each of a diverse genetic clade will enhance the ability to mitigate against the loss of adsorption or host genetic protection mechanisms. Genetic engineering may also provide a means to improve the diversity and targeting efficiency of phages for the avoidance of resistance (see Section 7). Another consideration is that bacterial mutations that confer phage-resistance often result in fitness costs to the resistant bacterium. Therefore, understanding and exploiting the fitness costs to resistant pathogens during therapy is a potentially promising research avenue [43].

## 6. Phage Therapy Clinical Trials in Humans

To date, human phage therapy trials have largely been empirical, with routine use limited to Georgia, Poland, and Russia [44]. In particular, the George Eliava Institute in Georgia has longstanding experience with the selection, isolation, and preparation of monophage and phage cocktails against a variety of bacterial pathogens for phage therapy. The therapeutic application of phages has also been undertaken for several decades at the Institute of Immunology and Experimental Therapy in Poland [45]. However, the experimental clinical data published in Russian and Polish journals are difficult to access due to security and language barriers.

Although the reporting and assessment of phage therapy need to improve, particularly with regard to efficacy and tolerability and the use of adequate patient numbers, several successful case reports have been published. The reports do provide some evidence that the development of phage therapy is a promising alternative to combat bacterial resistance to antibiotics.

In France, the national health regulator has authorized the first treatment of patients with extremely drug-resistant and difficult to treat infections using phage therapy. Since then, six cases with various bacterial infections have been successfully treated [44]. Even though several treatments were not conducted using clinical standards suitable for drug approval in the Western world, they showed therapeutic potential for phages and how phages can be applied [45].

**Figure 1:**
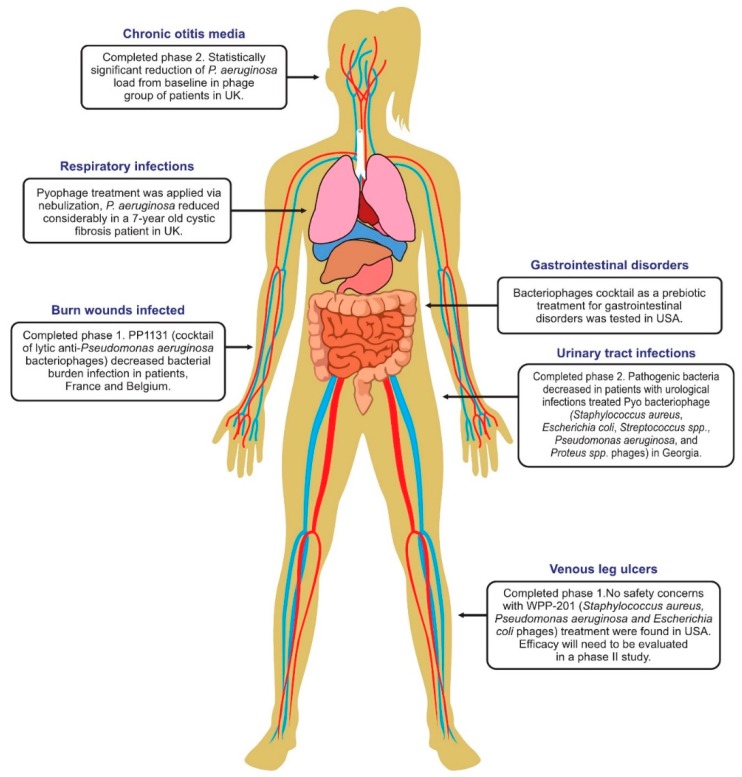
Human phage therapy trials and the range of target sites/infections. Image adapted from Furfaro et al. [46].

New therapeutic products must usually go through a long and comprehensive process involving preclinical and clinical trials to gain regulatory approval for market access. In the US, the average time for the approval of a new drug from preclinical testing is 12 years and the costs run into millions of dollars due to the length, size, and complexity of human clinical trials. For these reasons, the number of formal phage therapy clinical trials (as listed on www.ClinicalTrials.gov or https://globalclinicaltrialdata.com/) is very limited [45]. However, some of the human phage therapy clinical trials underway are summarized in Figure 1 and are described in the following case studies.

### 6.1. Phage Treatment of Burns

Phage therapy was applied in wound infections in 27 patients from hospitals in France and Belgium using a cocktail of virulent anti-*Pseudomonas aeruginosa* bacteriophages. Participants were randomly assigned (1:1) to a cocktail of 12 natural virulent anti-*P. aeruginosa* bacteriophages (10⁶ plaque-forming units [PFU] per mL) or standard of care (1% sulfadiazine silver emulsion cream), and the route of administration was topical for seven days, with 14 days of follow-up [44].

The median of the primary endpoint was 144 h in the phage treatment group and 47 h in the standard of care group. Three (23%) of the 13 analyzable participants showed adverse events in the phage treatment group when compared with seven (54%) out of 13 in the standard care group. Bacteria isolated from patients of the failed phage treatment were resistant to low phage doses [44].

This study showed that phage treatment decreased bacterial burden in burn wounds in more time than the standard treatment. In this regard, studies increasing the phage concentration and the use of “phagograms” (as used for antibiograms) with more patients are warranted.

### 6.2. Treatment of A Septicemia Patient with Acute Kidney Damage

A man in his sixties was hospitalized for *Enterobacter cloacae* peritonitis and severe abdominal sepsis, dispersed intravascular coagulation, herniation, and bowel strangulation. Following prolonged treatment for these ailments, the patient developed gangrene and pressure sores colonized by drug-resistant *P. aeruginosa*. The infection developed to septicemia and colistin treatment (the only drug sensitivity) was carried out, however, acute kidney damage was detected, and the treatment was suspended. Subsequently, phage therapy against *P. aeruginosa* was conducted using a mixture of two phages active against the isolate in vitro, under the umbrella of Article 37 (Unproven Interventions in Clinical Practice) of the Declaration of Helsinki [46]. Following phage therapy, the patient showed improved kidney function, which returned to normal function after a few days, and blood cultures were negative.

However, the patient’s pressure sores remained infected with *P. aeruginosa* and other species and four months later, the patient developed a refractory cardiac arrest due to blood culture-confirmed *Klebsiella pneumoniae* sepsis and the patient died. In vivo studies revealed that a *K. pneumoniae* strain isolated from the patient was sensitive to the antibiotics. According to historical reports, the use of phages by intravenous route in typhoid fever and *Staphylococcus aureus* [47] bacteremia were efficacious, nevertheless, this is the first contemporary report using phage monotherapy against *P. aeruginosa* septicemia in humans through the intravenous route [48].

### 6.3. Engineered Phages for Treatment of Mycobacteria in A Cystic Fibrosis Patient

Therapeutic phage treatment for mycobacteria has been explored in several animal models [49,50], but until recently had not been successfully used for mycobacterial infections in humans. A 15-year-old patient with cystic fibrosis and extensive comorbidities was referred for lung transplant with a disseminated infection of *Mycobacterium abscessus*. Following bilateral lung transplantation and persistent *M. abscessus* infections, phage genome engineering and forward genetics were used to engineer phages to target and kill the infectious *M. abscessus* strain. Therapy was conducted using an intravenous three-phage cocktail of 10^9^ PFU of each phage every 12 h for 32 weeks [51].

Intravenous phage treatment was well tolerated, clinical improvement including sternal wound closure, improved liver and lung function, and substantial resolution of infected skin nodules were detected in the six months following therapy. No evidence of phage neutralization was detected in sera, although weak antibody responses to phage proteins were identified. Weak cytokine responses were reported for interferon-γ, interleukin-6, interleukin-10, and tumor necrosis factor-α [51]. Some evidence was presented that indicated active in vivo phage replication was taking place. Despite the apparent success of this therapy, the authors did caution that there was significant variation in *M. abscessus* phage susceptibility, so the treatment of similar patients will require more work to be undertaken to understand the science underlying this observation.

### 6.4. Phage Therapy for Respiratory Infections

There have been several pre-clinical studies describing the use of phage therapy against chronic bacterial lung infections using murine models. Pabary et. al. [52] determined that phage treatment reduced the infective burden and inflammatory response in the murine lung. All phage-treated mice cleared *P. aeruginosa* infection at 24 h, whereas infection persisted in all of the control mice. Phage also reduced infection and inflammation in bronchoalveolar lavage fluid when administered prophylactically. Another study showed that intranasal treatment with phage rescued mice from *Acinetobacter baumannii*-mediated pneumonia. Microcomputed tomography also indicated a reduction in lung inflammation in mice given phage [53]. In a study using a biofilm-associated murine model of chronic lung infection, phage therapy was effective seven days post-infection. Additionally, these studies established the potential for phage therapy in established and recalcitrant chronic respiratory tract infections [54].

Notwithstanding the reported treatment of *Mycobacterium* described in Section 6.3, reports of phage therapy of human bacterial respiratory infections are rare. In a 2011 case study from Georgia, a seven-year-old cystic fibrosis (CF) patient presented with chronic colonization of *P. aeruginosa* and *S. aureus* with antibiotic treatments having limited effect. Phage therapy was undertaken using a “Pyo phage” phage cocktail produced by the Eliava Institute, which reportedly contains phages active against *S. aureus*, *Streptococcus*, *Proteus*, *P. aeruginosa*, and *E. coli* [55]. The Pyo phage cocktail was delivered to the patient via nebulization at four-to-six week intervals for nine rounds of treatment. The *P. aeruginosa* numbers reduced considerably, however, the treatment was not effective against *S. aureus*. Consequently, Sb-1 phage (a phage targeting *S. aureus*) was added to the Pyo phage cocktail and administered five times with a nebulizer. This treatment reduced the concentration of *S. aureus* significantly. No adverse effects were detected in the patient upon Sb-1 phage treatment [55].

Recent advances in the spray drying of phages that have achieve increased numbers of phages delivered to the lungs (up to 10^8^ PFU/aspiration) may considerably improve clinical outcomes for respiratory infections such as these [56]. Work has also shown that a cocktail of ten phages significantly decreased *P. aeruginosa* numbers in sputum samples from 58 CF patients collected from hospitals in Paris [45,57]. Forty-eight of 58 samples were positive for *P. aeruginosa* and the addition of phages significantly decreased the concentrations of *P. aeruginosa* in the sputum. An increase in the number of bacteriophages in 45.8% of these samples was also detected, demonstrating the potential for active phage therapy of respiratory infections in vivo.

### 6.5. Phage Therapy for Urinary Tract Infections

Therapy for treating urinary tract infections (UTIs) is one of the most promising applications for phages and one of the few that have been studied in a multi-stage clinical trial. In the first stage of the trial, 130 patients planned for transurethral resection of the prostate were screened for UTIs and 118 patients enrolled [58]. Criteria for inclusion in the trial were having ≥10^4^ cfu/mL of the pathogens *S. aureus*, *E. coli*, *Streptococcus*, *P. aeruginosa*, or *Proteus mirabilis* in their urine culture. Initial in vitro screening of these cultures against the Pyo phage cocktail, a commercial product produced by the Eliava Institute, revealed that the sensitivity was 41% (48/118). Directed evolution experiments were applied to the cocktail to select for expanded host range phages, and the sensitivity was improved to 75% (88/118).

In the second stage, nine patients who had infections caused by bacteria sensitive to the Pyo cocktail underwent non-blinded phage therapy. Administration of 20 mL 10^7^–10^9^ PFU/mL phages was via a suprapubic catheter twice every 24 h for seven days, starting the first day after surgery. Urine from the patients was subsequently cultured seven days after surgery or at the time of adverse indications. Prior to therapy, the patients’ urine screening revealed the presence of *E. coli* in four cases, *Enterococcus* in two cases, *Streptococcus* in two cases, and *P. aeruginosa* in one case. Following therapy, titers of the pathogens decreased by 1–5 log cfu/mL in six out of nine patients. One of the four *E. coli* cases had no detectable pathogen, one of two *Streptococcus* cases had no detectable pathogen, one of the *Enterococcus* cases had no pathogens, but the other case detected *E. coli*. The patient with the *P. aeruginosa* infection required antibiotic therapy following a spike in fever and became asymptomatic; however, *P. aeruginosa* was detected in his urine. No adverse effects of phage therapy were detected. The study authors hope to further progress this work to full randomized and blinded control studies in the future.

### 6.6. Phage Therapy for Diarrhea

Whilst no longer an active partnership, the Nestlé Research Centre in Switzerland and the International Centre for Diarrhoeal Diseases Research in Bangladesh have undertaken joint research projects over a number of years that have explored the efficacy of phage therapy for the treatment of diarrheal diseases. In one of the studies, 120 Bangladeshi male children (6–24 months) presenting with acute bacterial diarrhea were given either 3.6 × 10^8^ PFU of a T4-like coliphage cocktail (39 children), 1.4 × 10^9^ PFU of a commercial coliphage preparation (Coliproteus from Microgen, 40 children), or a placebo (0.9% NaCl, 41 children) suspended in oral rehydration solution.

Results of this randomized blind trial indicated no adverse effects of oral phage treatment of the children. The phage survived the gastric passage, but there was no strong evidence of intestinal replication occurring in patients. Neither the T4-like nor the Microgen coliphage cocktail showed a significant clinical effect when compared to the control group for stool output or frequency, or rehydration. Likely reasons for the lack of significant effects were the lower than expected incidence of *E. coli* (60%) and the incidence of mixed species infections, the presence of non-susceptible coliforms (phage cocktail was not optimized for local isolates), and insufficient phage titer [59,60].

### 6.7. Treatment of Peri-Prosthetic Joint Infection

In this case study [61], an 80-year-old female patient with obesity and a history of relapsing prosthetic joint infection of the right hip presented with a *S. aureus* postoperative infection and was treated with debridement, antibiotics, and implant retention (DAIR). Four years later, another DAIR was performed for fluoroquinolone-resistant *E. coli,* following reimplantation surgery in the prior year. Then, due to a relapse including positive *E. coli* cultures, another DAIR was performed three weeks later. Antibacterial therapy with ceftriaxone was started; however, there were further signs of relapse and antibiotic treatment was stopped. Multidrug-resistant *P. aeruginosa* and penicillin-resistant *S. aureus* were identified in swabs of the wound discharge.

To undertake phage therapy, three phages targeted against the *P. aeruginosa* isolate were first prepared by Pherecydes Pharma (France). The *S. aureus* isolate was lost, so three phages from the Pherecydes Pharma phage bank were used. Phages were produced in a research environment with the manufacture overseen by The French National Agency for Medicines and Health Products Safety (ANSM). The final formulation of the *P. aeruginosa* and *S. aureus* phages were undertaken by the hospital pharmacy by mixing equal volumes of 10^10^ PFU/mL phage stocks. During the following DAIR, 20 mL of the phage cocktail was injected into the joint region. Co-therapy with antibiotics (daptomycin, amoxicillin, and clindamycin) was followed for the next six months without signs of *P. aeruginosa* or *S. aureus* infection. The patient later had a *Citrobacter* infection, which required DAIR, but once this was treated with Ciprofloxacin, no further infection was found in the joint (18 months post-phage therapy).

**Figure 2:**
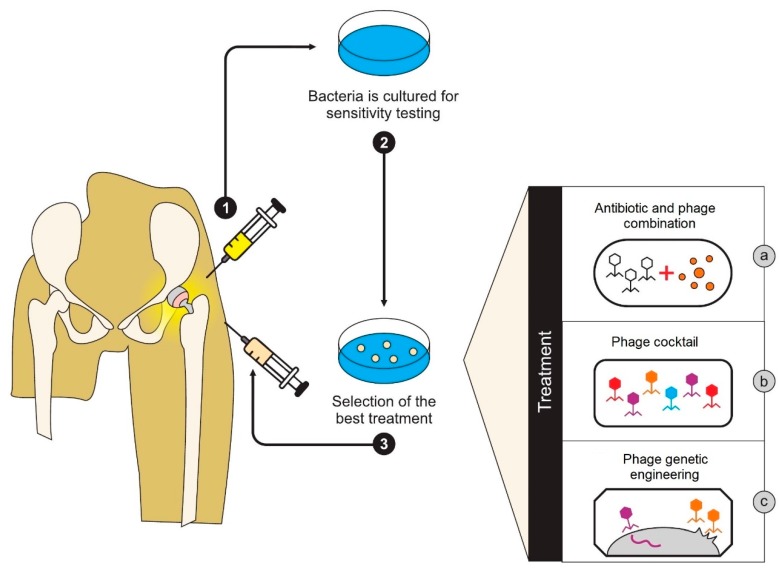
Personalized combinatorial phage therapy. Image adapted from Akanda et al. [62].

The bespoke use of phage and antibiotic combinations to treat a patient’s infection has the potential to be utilized to create personalized therapy for deep and persistent tissue infections such as those found associated with peri-prosthetic joints (Figure 2).

### 6.8. Treatment of Leg Ulcers

A Phase I trial of phage therapy with 42 patients with chronic venous leg ulcers has been undertaken [63]. Ulcers were treated for 12 weeks with a phage cocktail (WPP-201; 8 × 10^7^ PFU/mL) or a control (saline). The phage cocktail targeted *P. aeruginosa, S. aureus,* and *E. coli*. Patient follow-up continued until week 24 and no adverse events were attributed to the phage treatment. There was no significant difference between the phage therapy group and the control group for the rate or frequency of ulcer healing. Efficacy of the preparation will need to be evaluated in a phase II efficacy study.

### 6.9. Therapy of Drug-Resistant Craniectomy Infection

A previously healthy 77-year-old male who suffered assault, subdural hematoma, and traumatic brain injury underwent a craniectomy, which was complicated by postoperative intracranial infection with multidrug-resistant *A. baumannii*. The isolate was resistant to all antibiotics; however, some isolates were sensitive to colistin [64]. An emergency investigational new drug application to use phage therapy on the patient was approved by the US Food and Drug Administration. Phages from the Naval Medical Research Center-Frederick were screened against the isolate and the most active phage prepared.

The phage (2.1 × 10^7^ PFU/mL) was administered intravenously through a central catheter line every 2 h for eight days, with 98 total doses given. Following phage treatment, the patient initially seemed more alert, but continued to be unresponsive. The craniotomy site and skin flap healed well, though fevers and leukocytosis continued. There were no further signs of infection at the craniotomy site after surgical debridement. However, bacterial cultures obtained prior to phage administration were negative, therefore it was not possible to directly measure phage efficacy. Before the receipt of a second phage cocktail, the patient’s family decided to withdraw care and the patient died.

The authors concluded that administration of phages through the surgical drain would likely have had more benefit than parenteral administration, less targeted phages with broader activity may have been more efficacious, and a better outcome might have been possible if personalized phage therapy had been developed more quickly and administered earlier in the course of infection.

### 6.10. Therapy of Ear Infections

A Phase I/II research trial was conducted in the UK to test the efficacy and safety of phages for the treatment of chronic ear infections (otitis media), where the infection is known to harbor antibiotic-resistant *P. aeruginosa* [65]. In this randomized double-blinded study, a cocktail of six phages produced by Biocontrol Limited (BiophagePA, 6 × 10^5^ PFU), or placebo (glycerol-PBS solution) were administered to the ear canal of 24 patients. The follow-up to treatment was at 7, 21, and 43 days and revealed a statistically significant improvement in both clinical condition and patient-reported indicators for the phage treated group when compared to the control. No adverse reactions were noted in the phage-treated group.

In vivo replication of phages in the patients was evident for up to 23 days, with the mean recovery of phages during the trial sampling points being 200 times the input concentration. Clearance of phages was noted when the *P. aeruginosa* infection was resolved in patients. Reductions in overall *P. aeruginosa* numbers in the phage treatment group, whilst statistically significant, were generally modest, but measurement was likely to be compromised by the lack of access to deep parts of the ear canal. When *P. aeruginosa* was not completely cleared by phage therapy, there was an increase in clinical scores for some patients. The study authors suggest that repeated phage therapy after three to four weeks may be beneficial to these types of patients in any future work.

## 7. Engineering and Other Genetic Technologies for Phage Therapy

The advent of whole-genome sequencing and metagenomics have rapidly increased the number of phage genomes sequenced and is unlocking new insights into phage genetics. The use of this new knowledge for phage engineering holds great potential to increase the utility of phages for therapy, however, there are additional considerations such as ethical, safety, and regulatory, which need to be accounted for above that of ‘natural’ phage therapy. Engineering can be used to produce new variants of phages with expanded host range, decreasing the number of phage strains needed to cover bacterial diversity, and generating patentable phage variants [66,67,68]. For example, the host specificity of the *E. coli* K12-specific phage T2 was able to be changed by swapping gene products expressed at the tip of the long tail fiber with those of the PP01 phage, which is an *E. coli* O157: H7-specific phage [66]. The recombinant phage was able to infect *E. coli* O157: H7 and related strains, but could not infect *E. coli* K12 or its derivatives. Similarly, homologous recombination was used to replace the long tail fiber genes (genes 37 and 38) from the genome of T2 with those of the IP008 phage. The recombinant T2 phage had a host range identical to that of IP008 [67]. Lin et. al. [68] were also able to modify the *E. coli* female-specific T7 phage to overcome male exclusion by recombination with phage T3. The recombinant phages of T3 and T7 carried altered tail fibers and had better adsorption efficiency than T3.

Genetic engineering of phage permits the addition of novel functionality such as bacteriocins, enzybiotics, quorum sensing inhibitors, CRISPRs, and biofilm degrading enzymes that can enhance their killing potential [69,70,71,72,73]. Phages can be modified using the RNA-guided nuclease Cas9 to create sequence-specific antimicrobials. Cas9 was reprogrammed to target virulence genes and killed virulent, but not avirulent, strains of *S. aureus* in a mouse skin colonization model [65]. Another study used CRISPR-Cas technology to create RNA-guided nucleases delivered by phages to target specific DNA sequences in carbapenem-resistant *Enterobacteriaceae* and enterohemorrhagic *E. coli* [70]. Delivery of the nucleases improved the survival in a *Galleria mellonella* infection model. Phage-borne CRISPR-Cas systems can also be used to enable site-specific cleavage to induce cytotoxicity, activate toxin-antitoxin systems, re-sensitize bacterial populations to antibiotics, and modulate bacterial consortia [70].

Biofilms are the major cause of persistent infections in clinical settings, thus phage treatment to lyse bacteria in biofilms has attracted growing interest. An engineered T7 phage was constructed to encode a lactonase enzyme with broad-range activity for the quenching of quorum sensing molecules necessary for biofilm formation. The T7 phage incorporating the AHL lactonase *aiiA* gene from *Bacillus anthracis* degraded AHLs from diverse bacteria and caused the inhibition of a mixed-species biofilm composed of *P. aeruginosa* and *E. coli* [71]. In another approach using the T7 phage, a biofilm-degrading enzyme, DspB, produced by *Actinobacillus actinomycetemcomitans*, was inserted into the T7 genome and the resultant phage reduced *E. coli* biofilm cell counts by an additional 2 log when compared to the unmodified T7 [72].

As described in Section 4, components of the innate immune system can remove a significant proportion of administered phage. Studies have shown that long-circulating phage mutants can be isolated to address this issue. Vitiello et. al. [73] determined that a single specific substitution in the major phage capsid (E) protein of the lambda Argo phage was enough to confer a long-circulating phenotype that enhanced phage survival in the mouse circulatory system by more than a 1000-fold. Merril et. al. [74] used a serial passage selection method to isolate phage mutants with a greater capacity to remain in the circulatory system of the mouse. Lambda phage mutants with 13,000–16,000-fold better capacity to stay in the mouse circulatory system for 24 h after intraperitoneal injection were isolated.

Many antibiotics, as well as phage therapy, can present side effects due to endotoxin release from Gram-negative bacteria. To address this, genetic engineering was used to generate non-replicating non-lytic phage targeting *P. aeruginosa*. An export protein gene of the *P. aeruginosa* filamentous phage Pf3 was replaced with a restriction endonuclease gene and the variant (Pf3R) was non-replicative and prevented the release of phage from the target cell. Endotoxin release was kept to a minimum and the Pf3R phage efficiently killed a wild-type host in vitro. Phage therapy using Pf3R showed comparable or increased survival rates (depending on dose) when compared to Pf3 upon challenge in the mice model. Higher survival rates were correlated with a reduced inflammatory response when using Pf3R treatment [75]. Matsuda et. al. [76] also produced lysis-deficient T4 phages for this purpose. Mutant t amber A3 T4 phages were compared to wild-type T4 in mouse bacterial peritonitis model. Survival was significantly higher in mice treated with the lysis deficient phage when compared to the wild-type, and enterotoxin levels were significantly lower in the t A3 T4-treated mice at 12 hours after infection [76].

## 8. The Medicinal Regulatory Status of Phages

Phages are not specifically classified as living or chemical agents in any national medicinal legislation (as far as we are aware). This considerably complicates the regulation of human phage therapy clinical trials and commercialization of phage products as well-established safety, good manufacturing practice, and efficacy benchmarks are lacking [77]. Another barrier is that in order to prove the efficiency of phage preparations, their effectiveness and host range toward currently circulating pathogenic strains must be constantly monitored. This is most likely why the Russian Federation and Georgia approved phage preparations are continuously updated to target newly emerging pathogenic strains [78]. Therefore, any specific legislation regarding phage products would ideally permit these formulation updates as required to avoid repeated registration procedures.

A breakthrough for the regulation of phage therapy occurred in 2016, when the Belgian Minister of Social Affairs and Public Health defined the status of therapeutic phage preparations as industrially-prepared medicinal products (subjected to constraints related to marketing authorization) or as magistral (compounded) preparations prepared in the pharmacies’ officinal [79]. Natural phages and their products can be processed by a pharmacist as raw materials (active ingredients) in magistral preparations, providing there is compliance with several provisions of the European Directive requirements for medicinal products for human use [78].

Several jurisdictions also permit the use of phages on compassionate grounds, where all other therapies have failed, and the condition is immediately life-threatening. These include the US FDA Expanded Access Program (www.fda.gov/news-events/public-health-focus/expanded-access) and Investigational Drug Program (https://www.fda.gov/drugs/types-applications/investigational-new-drug-ind-application) and the European Medicines Agency (https://www.ema.europa.eu/en/human-regulatory/research-development/compassionate-use).

## 9. Advantages and Disadvantages of Phage Therapy

Compared to conventional antibiotic therapy for bacterial infections, phage therapy has both a number of great advantages, but also some disadvantages. Some of these have been summarized in Table 2 and some aspects are discussed in more detail in the following subsections.

### 9.1. Key Advantages

Phage therapy has several key advantages that make it an attractive alternative to antibiotics. First, phages have high specificity to their hosts and unlike antibiotics, which have a much wider spectrum, are unlikely to cause dysbiosis and secondary infections (e.g., fungal infections). To date, phages have also not shown any significant side effects or risks of toxicity on mammalian cells [80]. Moreover, the process of isolation and selection of new phages is less expensive, in terms of time and costs, than the development process required for antibiotics: it typically takes millions of dollars and numerous years to develop an effective antibiotic drug [81].

The development of the resistance of bacteria to phage therapy is likely less significant than for antibiotics because of the ability to adapt phage cocktails by the substitution of phages, applying in vitro evolutionary pressure, or by genetic engineering. The variant resistant mutants are also generally of lower fitness. Phages are also able to successfully treat multi-drug-resistant bacteria as they use different mechanisms for targeting cells.

The ability of phages to widely spread through the body when applied by systemic administration, along with self-replication in the presence of the host, are qualities that most antibiotics do not have. Unlike most antibiotics, phages can also pass through the blood–brain barrier [82]. Some phages can also infiltrate and disrupt the biofilms that many pathogens naturally inhabit [82,83].

For patients with allergies to antibiotics, their treatment options can be restricted. About 1% of hospitalized patients have an allergy to penicillin-group drugs, the most common antibiotic allergy, followed by sulfonamides and tetracyclines [84]. Cross-reaction of penicillin allergies to next-generation cephalosporins and carbapenems has also been reported, but this remains controversial [85]. Phage therapy may be a valuable option for patients with antibiotic allergies, but reports are rare. For example, 12 patients with inflammatory soft tissue shotgun wounds and allergy to antibiotics (not specified) were reported to have been treated by polyvalent phage therapy (*Staphylococcus*, *Streptococcus*, *Proteus vulgaris*, *Proteus mirabilis*, *P. aeruginosa*, *E. coli*, and *K. pneumoniae*) for 15 days [86]. The concentrations of bacteria and the areas of wound healing were similar in the phage treatment group when compared to a control group of 35 patients receiving antibiotics. The authors concluded that phages were a reliable method for reducing microbial infection and that treatment led to a rapid epithelialization of the wound site [86].

### 9.2. Key Disadvantages 

There are currently some key disadvantages of using phages as alternatives for antibiotics. However, these are predominantly due to gaps in knowledge and regulations, which may be resolved in the future. Critically, there is a lack of depth of information about the clinical application of phages for controlling bacterial infections. Much experimental clinical data published in Russian and Polish journals are difficult to access due to security and language barriers. There are also many more challenges for scientists in obtaining regulatory approval for phage-based therapeutic applications when compared to conventional therapies [80].

There is a lack of common established and validated protocols for the routes of administration, dose, frequency, and duration of phage treatment, which hampers inter-study comparison [87]. Often, the purity and stability of phage preparations used for clinical trials are also uncertain, with insufficient quality control data presented.

The concentration of phages may be reduced significantly during therapy by the reticuloendothelial system or be neutralized by antibodies, thus inhibiting their antimicrobial activity [39,88]. However, the effect of phage-neutralizing antibodies can be mitigated by refining dosing regimens and breeding phages to evade the immune system.

The genetic biosafety of phages is complex to assess. Phages used for therapy must not contain toxin or virulence genes, antibiotic resistance genes, or be able to horizontally transfer genes in the human microflora. Whilst whole-genome sequencing is a powerful tool to assist with these analyses, there is still an incomplete understanding of the functions of all encoded phage genes. Genetic engineering of phages will also likely invite greater scrutiny of safety which practitioners will need to address before application.

## 10. Conclusions 

Antimicrobial resistance is increasing globally, and new treatments are urgently needed to meet this challenge in medical care. Whilst phage therapy for bacterial infections has been around for more than a century, the antibiotic-resistance crisis is providing renewed impetus for phage therapy to deliver on its long-held promise as a clinical treatment. As described here, there is an increasing number of well-executed Phase I/II clinical trials describing the safety and efficacy of phage therapy. There is an improved understanding of the pharmacology, immunology, safety, and potential for bacterial resistance. Technologies such as genetic engineering, whole-genome sequencing, and metagenomics also provide new tools to optimize phage therapeutic strategies. However, there are still data gaps on its efficacy and a lack of standardization and suitable regulatory frameworks that need to be resolved before phage therapy can take its place in mainstream medicine. Given the renewed interest and impetus in the field of phage therapy, there are reasons to be optimistic that these challenges can be met in the coming years.

## Figures and Tables

**Table 1:** Routes of administration for phage therapy.

Delivery Route	Advantages	Disadvantages	Mitigations to Hurdles
Intraperitoneal	Higher dosage volumes possible. Diffusion to other sites.	Extent of diffusion to other sites may be overestimated in humans (most data from small animals).	Multiple delivery sites.
Intramuscular	Phages delivered at infection site.	Slower diffusion of phages (possibly). Lower dosage volumes.	Multi-dose courses. Multi-dose courses.
Subcutaneous	Localized and systemic diffusion.	Lower dosage volumes.	Multi-dose courses.
Intravenous	Rapid systemic diffusion.	Rapid clearing of phages by the immune system.	In vivo selection of low-immunogenic phages may be possible.
Topical	High dose of phages delivered at infection site.	Run-off from target site if phages suspended in liquid.	Incorporate phages into gels and dressings.
Suppository	Slow, stable release of phages over long time.	Limited applications/sites. Risk of insufficient dosing. Technically challenging to manufacture.	Careful consideration of phage kinetics required.
Oral	Ease of delivery. Higher dosage volumes possible.	Stomach acid reduces phage titer. Non-specific adherence of phages to stomach contents and other microflora.	Add calcium carbonate to buffer pH. Microencapsulation to deliver phages to target area.
Aerosol	Relative ease of delivery. Can reach poorly perfused regions of infected lungs.	High proportion of phages lost. Delivery can be impaired by mucus and biofilms	Use of depolymerases to reduce mucus.

**Table 2:** Advantages and disadvantages of phage vs. antibiotic therapy for the treatment of bacterial infections.

Consideration	Antibiotic Therapy	Phage Therapy
Specificity	Low	High
Development costs	High	Low-moderate
Side effects	Moderate-high	Usually low, but yet to be fully established
Resistance	Increasing incidence of multi-drug resistant isolates.	Can treat multi-drug-resistant isolates. Phage resistant isolates generally lack fitness.
Delivery to target	Moderate	Moderate to good. Can penetrate the blood-brain barrier.
Formulation	Fixed	Fixed or variable
Regulation	Well established	Underdeveloped
Kinetics	Single hit	Single hit or self-amplifying
Immunogenicity	Variable	Likely low, but not well established
Clinical validation	Many trial studies	Relatively few trial studies
